# Progress in Surgery

**Published:** 1897-11-20

**Authors:** 


### Progress in Surgery.

### INJURIES OF THE SPINE.

While injuries of the spine are by no means rare in hospital practice, it seldom falls to the lot of one surgeon to meet with any considerable number of such cases in a short period. Hence it comes that our knowledge of these conditions is the result of comparing and compiling the records of a large number of cases published by different observers, and therefore studied from various points of view. There has been recently published,[1] however, a paper by Poller, in which he analyses a series of 32 cases of vertebral fracture, met with by him during the previous five years. Such an experience is altogether exceptional, and the results of his investigations cannot but be valuable. We make it the basis of a brief *résumé* of recently published work on this subject.

**Cause.**—In the majority of Poller's cases the fracture was produced by the patients being bent forcibly forwards by falls of stone in mines—"shutting them up like a pocket-knife," as he puts it. Bending backward, direct violence and falls, were less common causes. Lobingier[2] reports a case in which a fracture dislocation of the second cervical vertebra was produced by muscular effort. The patient was an experienced athlete, and while performing on the horizontal bar he suddenly fell to the ground dead. No sound as of breaking bone or tearing ligaments was heard by the spectators, who were further satisfied that the patient struck no part of the structure on which he was performing. Post-mortem examination showed that there was no intra-cranial lesion, but the second cervical vertebra was found displaced forward and to the right for fully an eighth of an inch, the anterior common ligament being torn through. The adjacent lips of the second and third cervical vertebræ were broken, the fracture involving the right inferior articular facet of the second vertebra. The cord was soft for about an inch of its extent opposite the displaced bone. The author believes that the lesion was produced by a powerful contraction of the left posterior group of cervical muscles, with simultaneous and co-ordinate relaxation of the right anterior group.

**Lesions and Symptoms.**—In accordance with general experience, Poller found that the lesions were usually in those parts of the spinal column where a more and a less moveable portion meet, namely, in the upper cervical and lower dorsal regions. The physical signs present were more or less distinct hæmatoma at the seat of fracture, irregularity or deficiency in the line of the spinous processes; crepitus and mobility were much less frequently elicited. Interference with the functions of the bladder and rectum, priapism, disturbance of the heart's action and of the body temperature, and a form of diabetes mellitus were observed in different cases.

A case of a most unusual kind is reported by A. M. Watts,[3] in which the spinal cord was partially ruptured although the spine was not fractured. The patient, a man of 56, was thrown out of a cart. He walked to his home a mile off, and three hours after the accident rapidly lost the power of his hands and legs. There was later a loss of all sensation below the level of the third corsal cartilage as well as on portions of the upper limbs. There was entire loss of reflexes. He died of œdema of the lungs twelve days after the accident. On post-mortem examination it was found that there had been no gross lesion of the spinal column or of its ligaments; but the cord was found torn across on the left side at the junction of the sixth and seventh cervical vertebræ, and softened and degenerated throughout for a distance of about half an inch. The dura mater was torn at the same level. It is interesting to note that the reflexes were entirely lost, although the cord was not completely torn across.

In a case, recorded by Hudson,[4] where the fifth cervical vertebra was fractured, in addition to the ordinary symptoms, the author observed frequently on digital examination of the rectum, that there was *a total absence of contraction of the anal sphincter*. This he believes to be the only reported case of this condition.

**Diagnosis.**—As a rule, in Poller's cases there was no difficulty in diagnosis, the classic symptoms being present. A point sometimes raising doubt was whether the spinal arches or the bodies were involved. If the former the patient is usually able and anxious to sit up in a few days. If the pressure symptoms develop at once it usually is found that they are due to direct and primary crushing; if they come on in the course of a few hours or days hæmorrhage or inflammatory material is most likely their cause.

**Prognosis.**—This, of course, depends on the extent of the damage to the bone and cord. Poller found that when only the spinous processes and posterior part of the arches were damaged perfect recovery took place. One case in which priapism and two in which paralysis of the rectum and bladder were present recovered. Of 19 cases in which the spinal cord was implicated, 13 died, and 6 recovered more or less completely. Death in the fatal cases was usually traceable to sepsis in the urinary organs.

**Treatment.**—The author (Poller) favours treatment by immediate reduction of displacement under chloroform. While assistants make strong and steady traction from the head and feet, the surgeon manipulates the distorted portion of the spine into position. In some cases the bones could be felt to slide or spring into apposition. In 22 of his patients this method was adopted, without in any case being followed by paresis or anæsthesia. In one case where motion and sensation were abolished, the reposition of parts immediately restored these functions. He lays stress on the importance of keeping patients for at least two months absolutely at rest in bed, great care being taken to prevent bedsores or infection of the bladder when the catheter is necessary. After the bones have united, traction is made daily on the spine, and when this extension can be borne for half an hour at a time, a plaster jacket is applied and the patient allowed to use crutches. It is advised not to hurry patients out of bed too soon, as slow inflammatory conditions are apt to be set up, followed by deformity. Electricity, massage, and douches are of value in keeping up the tone of the muscles. By adopting this line of treatment, operation can often be dispensed with.

The results of operative interference in cases where a diagnosis cannot with certainty be made between compression and complete rupture of the cord, or in cases where less heroic measures have failed, are, on the whole, satisfactory. Chipault collected 164 cases of fracture operated upon, with 12 cases cured, 26 improved, 46 not improved, and 80 fatal. Lloyd gives the mortality at 25 per cent. So far as the operation itself goes, the only danger is sepsis, hæmorrhage and shock being much less serious risks than was formerly supposed. By careful manipulation the cord can be freely handled without harm resulting.

A recent case of laminectomy, published by Mayer, is of special interest, inasmuch as the operation was performed sixteen months after fracture with a successful issue. The record is also interesting as a specimen of Americanese. The patient, aged 37, was engaged on an elevator "which refused to work, and several attempts to stop it were futile." In attempting to leave it he "was crushed between the platform of elevator and ceiling of basement." He had the ordinary clinical signs of pressure on the cord in the upper lumbar region, followed by sepsis of the bladder, bed sores, and anklosis of the joints of the lower limbs. Ten months later, under chloroform, an attempt was made to move the hip joints, but, says the author, "despite the exertions of both (*sic*) Dr. Barbat, myself, and a nurse, only to a slight degree could the right leg be bent." On the left side similar manipulation "resulted in a vertical fracture of the patella." Three weeks later the process was repeated "with a little more success," and "Hoppe's patent extension splints were applied, to be used as a means to prevent re-adherence of adhesions." In time the cystitis was much improved, "incontinentia urinæ and fæces still persisting." Not content with the progress made, an operation was performed sixteen months after the injury, and the "laminæ of the first and second lumbar vertebræ showed a decided recedence from their preceding respectively following laminæ." Beyond an adhesion between the dura and the cord no other lesion was found. Progress was in every way satisfactory, and the patient was ultimately able to resume his work.

Hudson[5] reports a case of fracture of the fifth cervical vertebra, in which laminectomy was performed, and revealed the body of the fifth vertebra projecting backward so as almost completely to occlude the spinal canal. Death resulted eight days after operation.

Four cases of recovery after severe spinal injuries, without operation, were shown at the New York Academy of Medicine by Dr. Bailey,[6] but particulars are meagre in the report before us.

**Traumatic Disease of the Vertebræ.**—Henle[7] describes a condition which frequently follows traumatic lesions of the spine, based on six cases observed by him. The nature of the injury varies—a direct blow, a fall on the back or shoulders, or a forcible bending of the body. The symptoms at first are slight—pain in the injured region, with slight and transient anæsthesia and pareses. Later on, severe pain in the affected region comes on, sometimes with intercostal neuralgia, and interference with motion in the lower limbs; there is also a kyphosis with a projection in the back. The general health of the patient is seriously impaired. Although the kyphosis can be reduced, the "gibbosity" persists. The disease apparently consists in a softening of the bone from interference with its vitality, followed by the deposit of a soft callus, which yields to the pressure and weight of the higher part of the spine. The condition has to be diagnosed from tuberculous disease, gumma, tumour, and traumatic arthritis deformans. It is to be treated by rest in bed, with extension, or by mechanical supports. Kermisson[8] refers to a similar condition.

[1] Langenbeck's Archives, Bd. liv., Hft. 2. [2] Colorado Medical Journal, Feb., 1897. [3] Lancet, April 3, 1897; Brit. Med. Journ., March, 1897. [4] Journ. Nerv. and Mental Dis., June, 1897. [5] Annals of Surgery, Aug., 1897. [6] Med. Record, May 1, 1897. [7] Langenbeck's Archives, Bd. lii., Hft. 1. [8] Revue d'Orthopédie, 1896, No. 6.

